# Interstitial Fluid Behavior and Diseases

**DOI:** 10.1002/advs.202100617

**Published:** 2022-01-02

**Authors:** Wen‐Tao Liu, Yu‐Peng Cao, Xiao‐Han Zhou, Dong Han

**Affiliations:** ^1^ CAS Center for Excellence in Nanoscience National Center for Nanoscience and Technology Beijing 100190 P. R. China; ^2^ School of Future Technology University of Chinese Academy of Sciences Beijing 100049 P. R. China

**Keywords:** interstitial stream, substance transport model, efficient connection, imaging behavior omics, medicine of soft matter

## Abstract

Living things comprise a typical hierarchical and porous medium, and their most fundamental logical architectures are interstitial structures encapsulating parenchymal structures. The recent discovery of the efficient transport mechanisms of interstitial streams has provided a new understanding of these complex activities. The substance transport of interstitial streams follows mesoscopic fluid behavior dynamics, which is intimately associated with material transfer in nanoconfined spaces and a unique signal transmission. Accordingly, the evaluation of interstitial stream transport behavior at the mesoscopic scale is essential. In this review, recent advances in physical and chemical properties, the substance transport model, and the characterization methods of interstitial streams at the mesoscopic scale, as well as the relationships between interstitial streams and disease are summarized. Interstitial stream transport can be used as a basis to fully mine hierarchal behavior in images to expand imaging behavior into an omics field. By starting from the perspective of soft matter, a new understanding can be gained of health and disease and quantitative physical markers for research, clinical diagnosis, and treatment can be provided, as well as prognosis evaluation in complex diseases such as cancer and Alzheimer's disease. This will provide a foundation for the development of medicine of soft matter.

## Graphical abstract

The interstitial stream outlines horizontal fluid connection pathway based on the interstitial structure between various organs and tissues. The substance transports of the interstitial stream, including material transfer, energy transduction, and information transmission, serve to enable flat connections between functional structures in the human body and balance the healthy state of the human body.

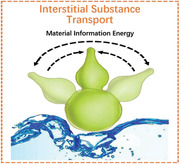

## 1 Introduction

In 1991, the Nobel laureate and French physicist, P. G. De Gennes,^[^ ^1^ ^]^ proposed the concept of soft matter and started promoting the interdisciplinary development of a subject that encompasses physics, chemistry, and biology. Complex living things show a hierarchical organization from molecules, cells, tissues, organs, and systems to an organism. From the material perspective, living things can be described as being composed of soft matter, such as intracellular stress fibers filled with intracellular fluid at the molecule level, a fibrous network filled with tissue gel in the extracellular matrix at the cellular level, capillaries at the tissue level, multi‐tissue organs and multiphasic media in these tissues at the organ level, and cavities and tissues at the organism level.^[^ ^2^ ^]^ Among these types of matter, multiphasic fluids in living things show an orderly multiscale spatial flow that determines biological activities. In biological systems, an orderly fluid flow is present in blood vessels and the urinary tract at the millimeter scale and in proton channels at the nanometer scale. These fluids comprise vertical connections in various parts of a living thing. In the circulatory system, the heart pumps blood to various organs and tissues in the human body, constituting vertical connections between organs and tissues. There is reason to believe that different fluid connections are present at all scales between millimeters and nanometers. Various levels of interstitial structure, which are distributed between tissues, constitute a path for fluid connections between these tissues. The concept of interstitial stream was proposed by Han et al.^[^ ^3^ ^]^ to outline this horizontal fluid connection pathway based on the interstitial structure between various organs and tissues. The substance transports of the interstitial stream, including material transfer, energy transduction, and information transmission, probably serve to enable flat connections between functional structures in living things and form a horizontal bridge between various parts of a living thing to integrate function and behavior. Hence, multiscale fluid behavior in interstitial structures is key for examining and understanding behavioral activities in living things.

## 2 Interstitial Structure and Interstitial Fluid

### 2.1 Definition of Interstitial Structure and Interstitial Fluid

The interstitium, which is present between parenchyma, is ubiquitous in complex living things. Collagen, elastin, and mucopolysaccharides connect to form a non‐liquid system consisting of a fibrous network filled with hydrophilic, porous media. This system is the interstitial structure, and the liquid in the interstitial structure is termed interstitial fluid.^[^ ^2^, ^4^ ^]^ Blood is a type of general connective tissue made up mostly of erythrocytes. Mature erythrocytes do not contain a nucleus and are rich in proteins. Plasma and mature erythrocytes can be viewed as a special type of interstitium in blood. When flowing, erythrocytes and plasma can act as a mobile fibrous network complex that is dispersed in blood. When blood remains static, erythrocytes and plasma tend to aggregate, forming a static fibrous network gel structure. Leukocytes and other cells are dispersed in this interstitium.^[^ ^5^ ^]^ Lymph is the convergence of interstitial fluid, that is, interstitial fluid from the mesoscopic state to macroscopic flow. In this sense, lymph and macromolecules constitute an interstitium.^[^ ^6^ ^]^ Currently, the tissue embryo model in biomedicine uses cells as the basic structure‐function unit. From this perspective, the interstitium is considered an extracellular matrix structure at the cellular level, and a fibrous network with matrix components in connective tissues at the tissue level.

In‐depth examination of past definitions of interstitial structure, connective tissue, loose connective tissue, dense connective tissue, and fascia tends to cause some confusion as even identical structures may have different names. As a result of current bias, the interstitial structure, which should be considered in its entirety, is separated into unrelated parts. Nevertheless, this can be resolved by considering the interstitium from the perspective of soft matter theory. The structure‐behavior‐function unit is the most basic in the human body, which is also the simplest form of fibrous network plus gel. All tangible structure‐function units and groups are bathed in this unit. The presence of hierarchal structures results in efficient interstitial connections. From the high porosity of fibrous networks (such as the extracellular matrix and loose connective tissue) to gradually denser fibrous networks, the matrix gel becomes compressed between single fibrous layers. Multiple layers are ultimately assembled into the fascia, which is compressed into tendons or is wrapped into a fascial space. This systemic interstitial structure, with its flattening connections, provides the most realistic image of biological activities, i.e., a “hierarchical complex fluid‐interstitial connection”. The entire human body is a hierarchical porous medium, in which cells are the characteristic functional structure units, tissues and organs comprise the next level of the functional structure units, and, ultimately, these units are dependent on flat interstitial connections to bond into a complete organism. As interstitial research progresses, the study of interstitial fluid attracts an increasing amount of attention.^[^ ^2^, ^3^, ^7^, ^8^, ^9^, ^10^, ^11^ ^]^

**Figure 1:**
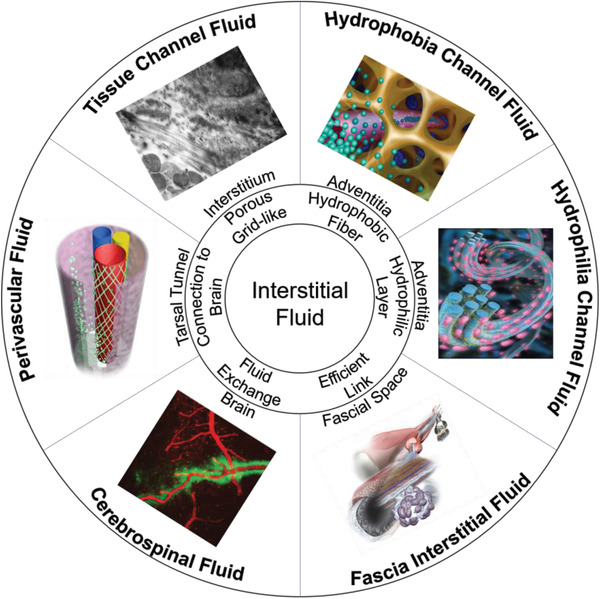
Display of the interstitial fluid, including tissue channel fluid, hydrophobia channel fluid, hydrophilia channel fluid, perivascular fluid, cerebrospinal fluid, and fascia interstitial fluid. Image for tissue channel fluid: Reproduced with permission.^[^ ^17^ ^]^ Copyright 1980, Elsevier. Image for hydrophobia channel fluid: Reproduced with permission.^[^ ^2^ ^]^ Copyright 2014, Springer Nature. Image for hydrophilia channel fluid: Reproduced with permission.^[^ ^8^ ^]^ Copyright 2017, IOS Press. Image for perivascular fluid: Reproduced with permission.^[^ ^3^ ^]^ Copyright 2019, Springer Nature. Image for cerebrospinal fluid: Reproduced with permission.^[^ ^10^ ^]^ Copyright 2012, AAAS. Image for fascia interstitial fluid: Reproduced with permission.^[^ ^11^ ^]^ Copyright 2018, Springer Nature.

Interstitial fluid is composed primarily of bulk water. Various cells, biological macromolecules, micromolecules, and ions can diffuse through the interstitial fluid or undergo directional flow.^[^ ^12^ ^]^ The situation is reversed in dense connective tissue as collagen fibers and elastic fibers are densely arranged such that almost all free water is expelled, leaving water that is immobile and bound to proteins. Multiple fibrous layers (usually three layers) are arranged in different directions to form the fascia, and the free diffusion or directional flow of interstitial fluids is inhibited by this fascia.^[^ ^13^ ^]^ Loose connective tissues^[^ ^14^ ^]^ lie between these two extremes as fibers are not densely arranged but are loosely connected to form a porous medium scaffold that is filled with interstitial fluid. In addition to bound and free water, the other components of the interstitial fluid in these tissues mostly show fluid behavior in nanoconfined spaces at the mesoscopic scale.^[^ ^15^ ^]^ **Figure** **1** shows interstitial fluid.

According to conventional knowledge, parenchymal structures that confer biological functions are encapsulated by the interstitium. The interstitial structures have been shown to have auxiliary functions such as support, protection, and segmentation, but their physiological and pathological functions and their significance have not received sufficient attention in research to date. Many studies^[^ ^2^, ^3^, ^10^, ^16^ ^]^ have examined interstitial fluid in recent years and have obtained important and inspiring results. Although our understanding of interstitial structures is clearer now, there is insufficient knowledge pertaining to interstitial fluid behavior. As more in‐depth research is conducted on interstitial fluid behavior, a greater understanding of its physiological function and significance in terms of complex diseases can be achieved.

### 2.2 Fluid Behavior in the Interstitial Structure

The free diffusion and flow of cells, molecules, ions, and nanoparticles in interstitial structures is usually narrowly defined as fluid behavior. This type of fluid behavior is intimately associated with material transfer in living things. It has an essential significance for understanding the physiological function of the interstitial structure and using this interstitial structure as a novel drug delivery route. In addition, it has become the focus of recent studies on interstitial fluid.

In 1978, Smith et al.^[^ ^7^ ^]^ employed the electron microscope to study lymphedema and found that plasticizers can enter micropores in the interstitial structure through the capillary network. Smith et al.^[^ ^17^ ^]^ were also the first to report the presence of conduction channels between interstitial structure and tissues. These channels have a randomly distributed porous grid‐like structure, and Smith et al.^[^ ^17^ ^]^ termed the flow paths for materials, energies, and information transport in fluid‐rich tissue as tissue channels. Li et al.^[^ ^18^ ^]^ employed radionuclide to study its migration trajectory along the meridians, which was closely related to the vascular system but no to the nerves. In addition, Li et al.^[^ ^18^ ^]^ pointed out that the meridian might be unknown functions of known structures. Zhang et al.^[^ ^19^ ^]^ found low hydraulic resistance channels in miniature pigs, which was related to interstitial fluid, but the specific anatomical structure and mechanism remained unclear.

However, it was not until 2012, when Nedergaard et al.^[^ ^10^ ^]^ employed two‐photon imaging to observe fluid transport behavior in the para‐vascular spaces of intracranial blood vessels in mice and found direct evidence of exchange between cerebrospinal fluid and brain interstitial fluid, that scientific interest in research on interstitial fluid was reignited. Nedergaard et al.^[^ ^10^ ^]^ named this pathway the glymphatic pathway. In subsequent studies,^[^ ^20^, ^21^ ^]^ it was found that metabolic micro‐molecules produced in the cranium can enter the circulatory system through this pathway, thereby clearing brain parenchymal waste products. It was thought that the glymphatic pathway replaces the role of the lymphatic system in the brain. In the same year, Yao. et al.^[^ ^22^ ^]^ simulated the directional interstitial flow under the parallel nature of capillaries conditions and explained that in vivo interstitial fluid flow might constitute the mechanical environment of cells and play a significant role in guiding cell activities. Mediation of mast cells may be the key to qi generation, indicating a close relationship with interstitial fluid.

Unlike the findings of Nedergaard at al.,^[^ ^10^, ^20^ ^]^ we found that interstitial fluid exists in somatic parts of the human body in the presence of lymphatic vasculature. Our research team first reported that long‐range fluid transport behavior is present in connective tissues in the tunica adventitia of veins, and this long‐distance efficient transport pathway, mediated by porous media at the mesoscopic scale, was termed the interstitial stream.^[^ ^3^ ^]^ These findings expanded our knowledge on directional transport of interstitial fluid from the brain to the entire body.^[^ ^2^, ^8^ ^]^ Further studies^[^ ^11^, ^23^ ^]^ found that intervaginal space injection of nanogold particles showed an in vivo biological distribution that is completely different from that of intravenous injection, demonstrating the potential for delivery of nanodrugs through this pathway. In addition, injection of liquid metals through this path can be used for encapsulating, segmentation, and treatment of tumors. Li et al.^[^ ^16^ ^]^, after injecting fluorescent molecules into fingers in human cadavers, conducted cardiac compressions and observed that a subcutaneous liquid conduction route appeared in the forearm, which entered the intermuscular layer at the upper arm before being traced to the atrial appendage.

### 2.3 Fluid Transport Behavior at the Mesoscale

Fluid transport behaviors perform what appears to be magic at the mesoscale. For example, Zheng et al.^[^ ^24^ ^]^ observed oscillations in the friction force between water and carbon nanotubes and showed that these oscillations result from the coupling between confined water molecules and the longitudinal phonon modes of the nanotube. They thought this coupling could enhance the diffusion of water molecules. The interstitial structure is in accordance with the characteristics of a hierarchical‐multiphase‐porous medium. Hence, interstitial fluid displays significantly different behaviors in various mesoscale spaces. For instance, we found that fast molecular transport along a multiphase interface serves as a high‐performance “green pathway” in a micrometer scale hydrophobic fiber network filled with a nanometer scale hydrophilic porous medium.^[^ ^2^ ^]^ In terms of this hydrophilic fiber network filled with a nanometer‐scale hydrophilic porous medium, we observed an increase of up to 80% in the diffusion coefficient of a fluorescent tracer subject to mechanical force stimulation compared to simple diffusion.^[^ ^8^ ^]^ However, fluid flowing in blood at the mesoscale performs significantly differently from that at a larger scale. The behavior of blood is often over‐simplified as a homogeneous Newtonian fluid in large blood vessels neglecting the low proportion of interactions between red blood cells and vessel walls.^[^ ^5^ ^]^ However, blood flowing in micro‐vessels cannot be considered a homogeneous fluid because it contains a high concentration of suspended cells, principally highly deformable red blood cells, whose dimensions are close to blood vessel diameters. Therefore, the ratio of interactions between red blood cells and the vessel endothelial cells is large. The surface effect plays a key role in capillaries, in which the mesoscale endothelial surface layers play a lubricated role to aid the motion of red blood cells.^[^ ^5^, ^25^ ^]^

**Figure 2:**
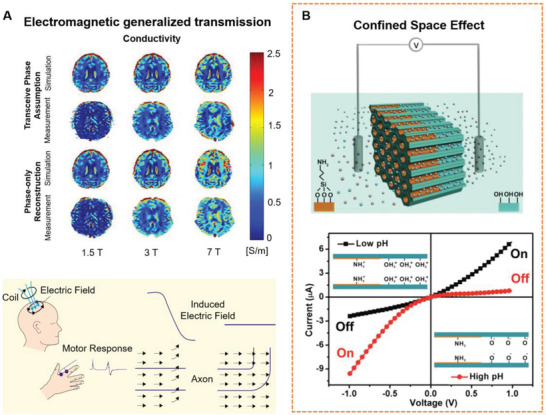
The substance transport phenomenon and mechanism. A) The transmission of current and magnetic fields is heterogeneous in the human brain. Image for current transmission: Reproduced with permission.^[^ ^27^ ^]^ Copyright 2014, John Wiley and Sons. Image for current transmission: Reproduced with permission.^[^ ^28^ ^]^ Copyright 2003, Elsevier. B) The nanoconfined space effect which enhances electric transporting. Reproduced with permission.^[^ ^26^ ^]^ Copyright 2013, Wiley‐VCH.

Not only is the performance of liquid transports fascinating, but electromagnetic transmission behavior within the human body is also intriguing. **Figure** **2** shows electrical and magnetic transmission in humans. Electrical and magnetic transmissions are heterogeneous in the human body. This heterogeneity is strongly associated with the interstitial structure and interstitial fluid behavior at the mesoscale. Regarding electric transport behavior at the mesoscale, Xia et al.^[^ ^26^ ^]^ found that the selected transport behavior of ions is established using highly anisotropic nanochannels of a porous anodic alumina membrane.

Material transfer and energy transmission at the mesoscale have been observed. Various physical signals are the carriers of information. Thus, information can also be transported at the mesoscale. The interstitial structure is a typical mesoscale structure. Therefore, we think that the transport behavior of interstitial fluid in the interstitial structure can take three forms, including material transfer, energy transduction, and information transmission. We summarized this as substance transport of interstitial fluid. Studies on the diagnosis and treatment of complex diseases, and the understanding and regulation of the interstitial stream have attracted increasing attention.^[^ ^27^, ^28^, ^29^ ^]^ Therefore, the substance transports of the interstitial stream have an important significance for understanding the physiological function of the interstitium and are crucial to the diagnosis and treatment of diseases. However, research on the substance transport of the interstitial stream is still phenomenological. A united mathematical‐physical model for the substance transport of the interstitial stream is needed.

## 3 Imaging Characterization and Modelling the Behavior of Interstitial Stream Substance Transport

### 3.1 Tensor Model for the Behavior of the Interstitial Stream General Substance Transport

In addition to interstitial fluid transport behavior being between the two extremes of free transport of bulk water at the macroscopic level and complete transport block in dense interstitial membrane structures, complex fluid state transport in multiscale porous media is also present. This has not been explored in relation to the traditional biological fluids (blood, lymph, and cerebrospinal fluid). Without loss of generality, the mixed interstitium can be divided into a fibrous scaffold plus gel complex (interstitial structure) and interstitial fluid at the microscopic level. From the analysis in the above section, it can be seen that the conduction coefficient of fluids does not have spatial direction selectivity, i.e., it is isotropic. When the microscopic arrangement of the fibrous scaffold shows a completely random distribution, the overall conduction coefficient of the interstitium is also isotropic.^[^ ^30^ ^]^ However, when the microscopic arrangement of the fibrous scaffold shows some directionality^[^ ^16^ ^]^ conduction in the interstitial structure can show macroscopic anisotropy.^[^ ^2^ ^]^ When this is the case, the interstitial fluid is affected by microscopic structural effects, and transport completely differs from macroscopic transport and is termed mesoscopic transport.

On the basis of previous studies,^[^ ^30^, ^31^, ^32^ ^]^ Torquato et al.^[^ ^33^ ^]^ proposed a valid transport model for anisotropic multiphase media. In order to describe the microscopic characteristics of the interstitial fluid, a set of tensor coefficients $A_{n}^{\left(i\right)}$ can be used for statistically quantitative characterization of microscopic structural information, which is defined as follows:

(1) $$\begin{array}{ccc} A_{n}^{\left(i\right)} & = & {\left(-1\right)}^{n}\varphi_{i}^{2-n}{\left(\frac{3}{4\pi }\right)}^{n-1}\;\int \int \;\cdot \cdot \cdot \int d{\vec{r}}_{2}d{\vec{r}}_{3}\cdot \cdot \cdot d{\vec{r}}_{n}t\left({\vec{r}}_{1}{\vec{r}}_{2}\right)\cdot t\left({\vec{r}}_{2}{\vec{r}}_{3}\right)\\ & & \cdot \cdot \cdot t\left({\vec{r}}_{n-1}{\vec{r}}_{n}\right)C_{n}^{\left(i\right)} \end{array}$$

where *ϕ*_*i*_ represents the volume fraction of the ith group, *U* represents the unit dyad, $t\left(\vec{r}\right)=\frac{d\vec{r}\vec{r}-r^{2}U}{r^{5}}$, and $S_{n}^{\left(i\right)}$ is the probability function for the nth point in the ith group; details on the parameter $C_{n}^{\left(i\right)}$ are available in a previously published paper.^[^ ^33^ ^]^ The conduction coefficient for the interstitial fibrous scaffold is *λ*_*e*_, the conduction coefficient for the interstitial fluid is *λ*_*i*_, the substance transport parameter Λ is a second‐order tensor in the three‐dimensional space, and spatial anisotropy is determined by the microscopic topology of the interstitial structure:

(2) $${\left(\varphi_{i}\beta_{\lambda }\right)}^{2}B^{-1}\left(\Lambda ,\lambda_{e}U\right)=-\sum_{n=1}^{\infty }A_{n}^{\left(i\right)}\beta_{\lambda }^{n}$$

where *B*(*X*, *Y*) = (*X* + 2*Y*)^−1^(*X* − *Y*) and $\beta \left(x,y\right)=\frac{x-y}{x+2y}$. Under normal circumstances, the substance transport coefficient Λ is a tensor, showing that mesoscopic transport has direction selectivity, i.e., the conduction efficiency is higher in some directions. When the volume fraction of an interstitial component decreases to zero or indicates that the tensor coefficient $A_{n}^{\left(i\right)}$ in microscopic topological information is isotropic, the substance transport tensor Λ in Equation (2) can be applied to bulk water.

Experiments have proven that the interstitial stream has a long‐distance efficient material transfer function.^[^ ^2^, ^3^, ^10^, ^11^, ^16^, ^20^, ^21^, ^23^ ^]^ These connecting pathways can be used for efficient and directional transport of fluorescent molecules, nanogold particles, paramagnetic contrast agents, and even liquid metals. The presentation of these novel fluid behaviors in interstitial structures is intimately associated with the unique fluid characteristics in nanoconfined spaces that have been discovered in recent years.^[^ ^34^ ^]^ Nanoconfined spaces show not only high fluidity for fluids but also high ion conductivity,^[^ ^26^ ^]^ demonstrating that interstitial stream pathways are general and efficient transport pathways for materials and information. Therefore, the general substance transport parameter Λ can be used to track efficient interstitial stream pathways. The spatial anisotropy of different spatial sites can be quantitatively characterized by using the fractional anisotropy (FA). The higher the FA, the higher the selectivity for this transport direction. The efficient interstitial stream pathway for interstitial fluid substance transport can be tracked by connecting the efficient transport directions throughout the space.^[^ ^35^ ^]^

Our model extended the mathematical‐physical description for liquid materials transport in the interstitial stream to materials, energy, and information transport behavior. A similar idea was used for the brain only.^[^ ^36^ ^]^ We extended the idea to encompass the whole body.

### 3.2 Available Potential Imaging Characterization of Transport Parameters

Traditional imaging methods have already elucidated the rudiments of substance transport behavior in interstitial streams. For example, diffusion magnetic resonance imaging (MRI) is currently the only non‐invasive imaging technique that measures the diffusion of water molecules in living tissue.^[^ ^37^ ^]^ Diffusion MRI aims at characterizing the water molecule diffusion of interstitial fluid, which has been used to track nerve fiber bundle interstitial stream pathways.^[^ ^38^, ^39^, ^40^ ^]^ However, interstitial stream imaging is rare. Non‐invasive imaging methods used in other fields have the potential for application in the characterization of substance transport in the interstitial stream.

**Figure 3:**
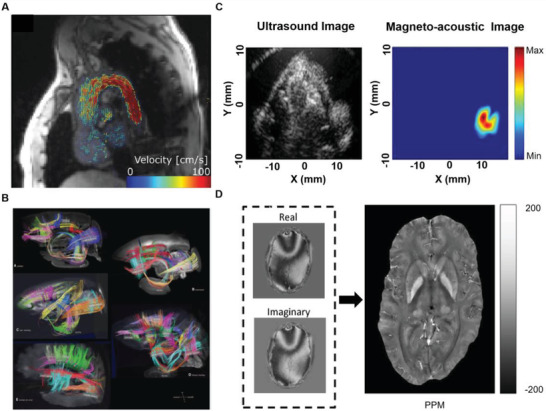
Potential characterization methods for interstitial stream substance transport in living things. A) Flow velocity imaging was used to characterize material transfer of fluid. Reproduced with permission.^[^ ^41^ ^]^ Copyright 2019, John Wiley and Sons. B) Diffusion imaging was used to characterize the spatial anisotropy of diffusion behavior. Reproduced with permission.^[^ ^40^ ^]^ Copyright 2012, AAAS. C) Magneto‐acoustic tomography showed high performance on characterizing the iron oxide nanoparticles concentration. Reproduced with permission.^[^ ^42^ ^]^ Copyright 2016, Elsevier. D) Quantitative magnetic susceptibility imaging was used to measure the spatial distribution of magnetic susceptibility in the human body. Reproduced with permission.^[^ ^43^ ^]^ Copyright 2016, John Wiley and Sons.

Flow can be measured by MRI, and corresponding MRI measurement techniques have also been developed for detection of electrical and magnetic characteristics. The newly emerged magneto‐acousto tomography has great potential in conductivity measurements. **Figure** **3** shows four types of imaging characterization used for transport parameters.

Flow velocity imaging: Phase‐contrast (PC) MRI^[^ ^44^ ^]^ can be used to measure flow velocity and direction. In recent years, PC‐MRI‐based 4D flow imaging^[^ ^45^ ^]^ has undergone rapid development and is widely used for cardiovascular hemodynamic assessment. Analysis of the flow velocity field can also be used to calculate other fluid dynamic parameters, such as pressure gradient and shear force.^[^ ^46^ ^]^ Davies et al.^[^ ^47^ ^]^ combined PC flow velocity coding and echo planar imaging sequences to increase the temporal resolution of flow velocity measurements. Electromagnetic imaging: In 2004, Leigh et al.^[^ ^48^ ^]^ proposed the concept of quantitative magnetic susceptibility imaging. After improvements were made by Haacke et al.^[^ ^43^, ^49^ ^]^, this technique could accurately measure the spatial distribution of magnetic susceptibility in the human body. By changing the angle direction between the test sample and the main magnetic field, the anisotropic magnetic susceptibility tensor can be used for imaging.^[^ ^50^ ^]^ Katscher et al.^[^ ^51^, ^52^ ^]^ used the second‐order derivative of the three‐dimensional amplitude and phase distribution of the radiofrequency field in MRI to quantitatively characterize the spatial distribution of the dielectric constant and conductivity in the human body. In recent years, emerging magneto‐acoustic tomography has been used to mutually convert electromagnetic signals and ultrasound signals, which can precisely measure conductivity distribution in organisms.^[^ ^53^, ^54^ ^]^

### 3.3 Prospective Imaging Characterization of Transport Parameters

Not all parameters of interstitial stream substance transport can be measured with current imaging methods. In addition to flow velocity, diffusion, electrical, and magnetic imaging, direct non‐invasive in vivo measurement and imaging are still not possible for other transport parameters (such as thermal conductivity, acoustic conductivity, bulk modulus, and elastic modulus). In addition, existing electrical properties imaging cannot be used to characterize the spatial anisotropy of the dielectric constant and conductivity. On the other hand, measuring magnetic susceptibility tensor requires multiple changes in the placement of the sample and has many limitations, particularly when used for imaging of the human body. In physics and engineering, the successful use of easily measured physical parameters to indirectly characterize physical parameters (that are difficult to measure) is also exceedingly difficult and problematic. Levin^[^ ^55^ ^]^ first linked the bulk modulus and effective thermal expansion coefficient in isotropic diphasic mixed media. Milton^[^ ^32^ ^]^ obtained the correlations between the effective dielectric constant, bulk modulus, and shear modulus in two‐component composite materials. Cherkaev and Gibiansky^[^ ^56^ ^]^ established the relationship between the dielectric constant and magnetic susceptibility. Milton^[^ ^57^ ^]^ proposed a universal method for establishing correlations among various parameters.

As mentioned in Section 3.1, understanding the anisotropy for transport behavior is crucial for research on efficient connection. Torquato et al.^[^ ^33^ ^]^ extended the parameter relationship to anisotropic two‐component media and used the tensor coefficient to characterize the topological characteristics of the microscopic structure. In 2001, Tuch et al.^[^ ^36^ ^]^ proposed using the diffusion tensor to indirectly characterize the conductivity tensor. They were the first to apply the association attributes of two parameters in imaging measurements. This method is currently used in brain conductivity distribution assessment before transcranial current brain stimulation.^[^ ^58^ ^]^ Frau‐Pascual et al.^[^ ^59^ ^]^ used this method to construct an electrical conduction model of brain structure connections. The idea was that association attributes can play an important role in substance transport imaging of interstitial stream. Based on the tensor model of interstitial stream substance transport, by using existing diffusion, flow, and electromagnetic imaging methods and constructing quantitative relationships between association attributes, we can indirectly characterize values and spatial anisotropy of transport parameters that are usually difficult to directly measure, such as thermal conductivity, acoustic conductivity, bulk modulus, and elastic modulus. This can provide comprehensive technical support for interstitial stream substance transport behavior studies.

## 4 The Relationship between Diseases and Interstitial Stream Substance Transport Behavior

With the emerging dominance of complex diseases such as cerebro‐cardiovascular disease, malignancy, and degenerative disease, it is difficult for the structure‐function‐based biomedical model to guide development of modern medicine, and there is a need for reorganization and a new understanding of the nature of biological activities. Currently, various types of information on fluid behavior in the human body are used for diagnosing disease. For example, in cerebro‐cardiovascular disease, MRI can provide images of different phases in the cardiac cycle to obtain the cardiac motion pattern and calculate local strain and related cardiac wall motion parameters, which are used to assess overall cardiac function and diagnosis of myocardial diseases.^[^ ^60^ ^]^ Many studies found that blood flow and perfusion behavior in the brain and functional and structural changes in blood vessels are important risk factors for many neurodegenerative diseases. Hence, MRI perfusion imaging is expected to provide imaging markers for early prediction of Alzheimer's disease.^[^ ^61^ ^]^ In addition to cerebro‐cardiovascular disease, blood flow in tumors is also an extremely important reference for cancer diagnosis. For example, dynamic contrast‐enhanced MRI is used to analyze and compare the spatiotemporal patterns of contrast agent entry/exit in tumor tissues and is used clinically for cancer detection, diagnosis, staging, and treatment response evaluation.^[^ ^62^ ^]^ Diffusion tensor imaging can be used to measure the diffusion tensor of water molecules in cerebral white matter,^[^ ^63^ ^]^ thereby tracking and imaging nerve fiber bundles.^[^ ^38^ ^]^ This method has been applied in the clinical diagnosis of Alzheimer's disease, demyelination, and stroke. In addition, information on force transmission in patients has also been applied in diagnoses for many years. For example, ultrasound elastography is already widely used in the diagnoses and treatment of liver, breast, and thyroid disorders and has achieved satisfactory results.^[^ ^64^ ^]^

The synergy of the whole body should be considered when treating complex diseases. Many studies have pointed out that there are significant correlations between different organ and tissue diseases, such as between chronic obstructive pulmonary disease (COPD) and intestinal bowel disease (IBD).^[^ ^65^ ^]^ Patients with COPD have a two‐ to three‐fold higher probability of being diagnosed with IBD.^[^ ^66^ ^]^ With developments in research on gut and lung disease correlations, the concept of the gut‐lung axis has been proposed.^[^ ^67^ ^]^ Similarly, there is also a large number of studies on the correlation between the gut and brain and between kidney disease and cardiovascular disease.^[^ ^68^ ^]^ Although many studies have examined the molecular mechanisms between diseases, the relationship between immune functions in these distal organ and tissue diseases has not been completely elucidated.^[^ ^67^ ^]^ Visceral fat tissue contains a large amount of interstitial structures. Studies have reported that intestinal bowel diseases and lung diseases can both change the inflammation status of visceral fat tissue.^[^ ^69^ ^]^ These results imply that interstitial streams are an essential link between multiple organs. Understanding the relationships between distant tissues throughout the whole body is crucial for the diagnosis and treatment of complex diseases.

Overly focusing on structure and ignoring fluid transport information, means that the synergy of the whole body may be underestimated. After discovering the long‐distance‐transport interstitial stream, our research group conducted a series of further studies. For example, preclinical studies on a mouse breast carcinoma in situ model found that intervaginal space injection of liquid metal can be transported to within the vicinity of the carcinoma in situ along the interstitial stream pathway.^[^ ^9^ ^]^ The aforementioned reports concluded that distal tissues in living things can form a long‐distance, direct, and efficient connection through the interstitial stream that is present throughout the body and is mutually connected.^[^ ^3^ ^]^ The fascial space at the macroscopic scale and the interstitial structure at the mesoscopic scale can transport a substance containing large amounts of materials, energies, and information, which provide a material basis for efficient and coordinated communication between various organs and tissues. Therefore, there is reason to believe that the interstitial stream enables flat connections between functional structures in living things through material, energy, and information connections and forms a horizontal bridge between various parts of the organism to integrate function and behavior. Herein, our tensor model of interstitial stream substance transport will significantly contribute to the completion of a full characterization of fluid transport methods, thus, facilitating the diagnosis and treatment of diseases.

The examples above demonstrate the positive roles of fluid behavioral information in disease diagnosis. Many methods for characterizing material, energy, and information transport used in clinical practice and research studies can be used to characterize interstitial stream substance transport behavior. The response pattern of biological soft matter to the external environment is non‐linear. Therefore, many static markers cannot wholly describe the status of tissues and organs. However, the studies described above acquired only a single or several markers and used superficial statistical differences to differentiate diseases. They also did not consider constructing links between characterization parameters and physical status. Our group used fluid mechanics as a base to establish links between behavioral information and biological status. We then proposed cell modulus measurement and dynamic fitting of pathological sections.

**Figure 4:**
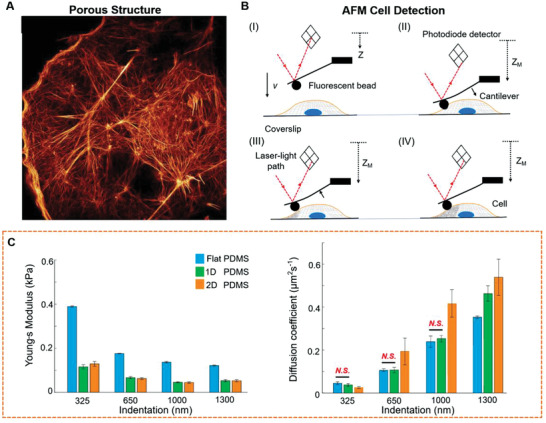
A) Image of actin in a cell showing that the cytoplasm can be treated as a biphasic material containing a porous elastic solid network immersed in an interstitial fluid. Reproduced with permission.^[^ ^73^ ^]^ Copyright 2015, AAAS. B) Graphic diagram showing measurement for cytoplasmic rheological parameters. C) Graphs of poroelastic and elastic properties altering in their responses to changes in cell volume. Reproduced with permission.^[^ ^74^ ^]^ Copyright 2019, AIP Publishing.

As a basic unit vital to living things, mechanical model analysis of single cells is an extremely active and challenging research domain. To describe and analyze the physical behavior of cells, researchers have developed many physical models using different perspectives. Based on the porous medium model, we analyzed intracellular fluid phase behavior and improved the data extraction method for the cellular Young's modulus to obtain a cell modulus measurement method closer to the cellular scaffold modulus. By integrating intracellular apparent viscosity^[^ ^70^ ^]^ and the cellular diffusion coefficient,^[^ ^71^ ^]^ we proposed a multiparametric method for characterizing the physical characteristics of cells. This method can distinguish between different types of cells and can also be used to classify and identify healthy cells and cancer cells of the same type. Compared with using Young's modulus alone to distinguish between healthy and cancer cells,^[^ ^72^ ^]^ this method is greatly superior. **Figure** **4** shows the porosity of the cell, the experiment scheme for poroelastic parameters, and the effectiveness of this approach.

Pathological diagnosis is currently the gold standard for disease diagnosis. We found that there are also considerable differences in the prognosis for different Tumor‐Node‐Metastasis (TNM) pathological stages. Our group started by using topographical and mechanical effects to analyze the relationship between the curvature of adenocarcinoma tissues and late‐stage invasion. We found that the greater the curvature of the tumor gland tissues, the greater the malignancy and the poorer the prognosis.^[^ ^75^ ^]^ Therefore, we proposed a dynamic fitting method for pathological section information and obtained the disease progression trend by fitting geometric pathological markers. This enabled a more accurate evaluation of the progress of patients with colon cancer.

The method mentioned above was used to construct relationships between behavioral information and disease state and prognosis progression trends at the cellular and tissue levels. The method was then used to propose a relevant mechanical and fluid‐related parametric model for prediction, which converted static medical information into dynamic behavioral descriptions. This method provided the idea for selecting the most informative type of information that can best reflect biological behavioral activities among numerous types of behavioral information. It provided a direction for constructing a relationship model that analyzed the substance transport behavioral information of the interstitial stream and biological status. In addition, as living things themselves naturally possess specificity, it is impossible to employ conventional statistical markers (mean, variance, median, and mode) to completely describe the substance transport of interstitial stream spatial distribution in the entire lesion. Therefore, we should integrate information on the structure and fluids of living things to expand the development of imaging behavior omics, particularly the physical/chemical/biological behavior of the interstitial stream. This would be a breakthrough in the development of appropriate new medical imaging and characterization methods and the construction of a mathematical model on imaging behavioral characteristics and disease, which would provide theoretical and technical support for accurate diagnosis and personalized treatment.

## 5 Problems and Challenges

Studies on the relationship between interstitial stream substance transport and human health are still in their infancy. Enormous problems and challenges remain to be solved. The physiological implications of interstitial stream transport behaviors are critical. Interstitial stream pathways are the basis of studies in this field. At present, only a few interstitial stream pathways between limbs, organs, and brains have been elucidated.^[^ ^9^, ^11^, ^23^ ^]^ More efforts are needed to depict interstitial stream pathways in the human body. In addition, the dynamic mechanism underlying the interstitial stream is still unclear. A large number of studies have shown that matter performs unique transfer characteristics in nanoconfined space.^[^ ^76^ ^]^ The multiphase and multiscale fibrous gel network in the human body contains abundant micro‐nanometer scale confined space. The force resulting from the evaporation of lung and sweat glands on the surface may be one of these dynamic sources. Muscular movement and vascular pulsation may also be dynamic sources for the interstitial stream. Hence the behavior of interstitial fluid substance transport in the human body is worth exploring.

The relationship between the occurrence, progression, and prognosis of a disease and the transport of interstitial stream substances under pathological conditions is critical. For example, in invasion and metastasis, tumor‐derived exosomes can induce liver pre‐metastatic niche formation to increase the metastatic liver burden.^[^ ^77^ ^]^ The role of the interstitial stream material transport in this process is very intriguing. In addition, the biological effects of interstitial stream energy transport on the immune system in the interstitial stream pathway are also worth studying. Mechanical action from interstitial fluids can stimulate the immune system.^[^ ^78^ ^]^ For instance, fasciitis in the interstitial stream pathway is an immune‐related disease,^[^ ^79^ ^]^ and the energy transport behavior of interstitial streams is likely to play a significant role in its development. Moreover, physical quantities can carry information and produce significant biological effects in the path of the interstitial stream, thus affecting the pathological process of human disease. For example, a warm‐sensitive neuron in the preoptic area can respond to thermo‐sensory signals.^[^ ^80^ ^]^ In conclusion, the substance transport behavior of the interstitial stream is closely related to the physiological states of human diseases. Clarifying the biological significance of the substance transport behavior of the interstitial stream will substantially promote medical science development.

Developing methods of imaging characterization are key to transforming research results on interstitial stream substance transport into medical applications. However, state of the art imaging characterization methods can only characterize part of the interstitial stream substance transport, such as fluid flow and diffusion, electrical conduction, and elasticity.^[^ ^36^, ^38^, ^39^, ^51^, ^54^, ^64^ ^]^ Many physical characteristics of the transport are difficult to characterize. Moreover, only a single direction can be detected, making it difficult to characterize the physical characteristics of the anisotropy. It is key that translational medicine develops a characterization method for medical imaging of physical quantities, detects more types of interstitial stream transport behaviors, and detects the transport characteristic direction of corresponding physical quantities. Magnetic‐acoustic‐electronic imaging, MRI, ultrasonic imaging, and Terahertz imaging have great potential for uncovering the substance transport behavior of the interstitial stream. To extend the above tools to measuring more transport parameters and tracking interstitial stream pathways, more work should be done to complete the tensor model of interstitial stream substance transport. After obtaining information on interstitial stream transmission behavior, it will be a significant challenge to rationally use this information for disease diagnosis and treatment. However, no perfect method for analysis of information on the substance transport behaviors of the interstitial stream has been proposed at present. Establishing an approach to bridge the relationship between interstitial stream substance transport behavior and disease state is critical to improving its medical translation.

## 6 Summary

Living things are typically composed of soft matter in a logical structure composed of various interstitial levels that are distributed among parenchymal tissues. The interstitial stream connects material, energy, and information to enable flat connections between functional structures in living things and form a horizontal bridge between various parts within the organism to integrate function and behavior. The tensor conduction model can describe the substance transport behavior of the interstitial stream in complex living things. It can provide a new way of understanding the complex activities within life forms, and potentially track interstitial stream pathways. The breakthrough of using the physical/chemical/biological behaviors of interstitial streams and integrating biologically complex fluid behavior allows the existing acquisition method for most static medical information to be converted to dynamic behavioral descriptions, such as the cellular modulus, dynamic fitting of pathological sections, ultrasound elastography, and MRI. This can be used to develop a new multiscale behavior‐related medical imaging and characterization method, to construct a mathematical model on imaging behavioral characteristics and disease, to provide theoretical and technical support for accurate diagnosis and personalized treatment, and to expand imaging behavioral omics. Imaging and characterizing substance transport behavior of interstitial streams in living bodies and organizing the links between various tissues and organs can inform systematic and quantitative research and provide a clinical application tool to diagnose complex diseases and formulate personalized physical therapies.

## 7 Outlook

In the last century, the invention of antibiotics and vaccines and the emergence of various advanced medical equipment has allowed genomics and biomedical research to gradually move from the body, organ, tissue, and cell level to the molecular level. This has had a massive impact on human health, and the healthcare industry and medical science and technology have improved dramatically. This has laid the foundation of a health view, disease view, and diagnosis and treatment view of structure‐function coupling. Although our understanding of diseases has deepened, and diagnosis and treatment of clinical diseases have undergone rapid development, the benefits of these medical developments on human health seem to be limited. With the emerging dominance of complex diseases such as cerebro‐cardiovascular disease, malignancy, and degenerative disease, the structure‐function‐based biomedical model overly focuses on the tangible structure, ignores dynamic information, and relies mostly on static objective markers. Its standards originate from statistical analysis of big data and the subjective judgment of physicians, which usually separates patients and diseases. Therefore, inaccurate disease definition causes a bias in treatment logic. Modern medicine should reference theories that conform to biological behavioral activities to improve disease definition, thereby guiding treatment logic to overcome the doctrine and limitations of the biomedical model and so become more effective. Starting from the soft matter theory, focusing on multiscale biological fluid behavior; re‐describing biological activities; conducting in‐depth research on the structure‐behavior‐function mechanism; employing methods such as medical imaging, clinical treatment, biological materials, new drug development, and non‐drug treatments; and compensating for shortcomings in actual clinical activities of the current structure‐function‐based “hard matter” thinking in mainstream medicine can provide a basis for the development of a new medical model, i.e., a PEO‐medicine (People‐Excitement‐Obedience Medicine) that is represented by the medicine of soft matter.

## Conflict of Interest

The authors declare no conflict of interest.
